# Effects of Supervised Rehabilitation on Psychosocial and Participation-Related Outcomes After Lumbar Spine Surgery: A Systematic Review and Meta-Analysis

**DOI:** 10.3390/jcm13237246

**Published:** 2024-11-28

**Authors:** Francesco Scandelli, Davide De Leo, Giorgia Marino, Emanuela De Martino, Delia Cannizzaro, Paola Adamo, Federico Temporiti

**Affiliations:** 1 Department of Biomedical Sciences, Humanitas University, Via Rita Levi Montalcini 4, 20072 Pieve Emanuele, Milan, Italy; 2 Physiotherapy Unit, IRCCS Humanitas Research Hospital, Via Manzoni 56, 20089 Rozzano, Milan, Italy; 3 Department of Neurosurgery, ASST Ovest Milano Legnano Hospital, 20025 Legnano, Milan, Italy

**Keywords:** lumbar spine surgery, supervised rehabilitation, psychosocial outcomes, participation-related outcomes

## Abstract

**Background/Objectives:** Supervised rehabilitation has been reported to improve motor and functional outcomes after lumbar spine surgery. However, the effects of supervised rehabilitation on psychosocial and participation-related outcomes are still debated. This study aimed to systematically review the effects of supervised rehabilitation on psychosocial and participation-related outcomes in patients after lumbar spine surgery. **Methods**: A systematic literature search was carried out using PubMed, EMBASE, CINAHL, PEDro, CENTRAL, and Google Scholar databases from inception to March 2024. Randomized controlled trials investigating the effects of supervised rehabilitation on psychosocial and participation-related outcomes after lumbar spine surgery were included. Methodological quality was assessed through the revised Cochrane risk of bias tool for randomized trials. Pooled effects were reported as the standardized mean difference (SMD) with a 95% confidence interval (CI_95_) or reported qualitatively in the presence of clinical heterogeneity. The certainty of the evidence was rated through the GRADE approach. **Results**: Fifteen studies (1297 patients) were included. Very low evidence supported supervised rehabilitation to improve quality of life at 1 year (SMD: −0.28; CI_95_ from −0.49 to −0.07, I^2^ = 32%), while low evidence supported supervised rehabilitation to enhance self-efficacy at 6 months (SMD: −1.13; CI_95_ from −1.54 to −0.72) and 1 year (SMD −1.03, CI_95_ from −1.43 to −0.63). No effects of supervised rehabilitation were found on quality of life at 6 months or in terms of fear-avoidance belief, catastrophizing, anxiety, depression, and return to work at 6 months and 1 year (very low to low evidence certainty). **Conclusions**: Supervised rehabilitation improved quality of life and self-efficacy in patients after lumbar spine surgery. However, the certainty of the evidence ranged from very low to low, and further studies are needed.

## 1. Introduction

Low back pain affects more than half a billion people worldwide [1], and the incidence is expected to grow in the coming years, contributing to increased disability and sick leave [2]. When considering patients with low back pain, symptomatic spinal stenosis, disc herniation, and spondylolisthesis are the most frequent clinical conditions leading to surgical referral [3]. Spinal surgery has been reported to be associated with high success rates and significant improvement in symptoms at 1 year [4,5]. However, a decrease in quality of life and the onset of psychological issues, including low self-efficacy, high fear-avoidance beliefs, and catastrophizing, have been reported in patients after lumbar spine surgery. These psychological factors negatively influence the intensity, duration, and prognosis of residual symptoms [6,7].

Data in the literature have reported that supervised exercises are beneficial in improving pain and disability in patients after lumbar spine surgery [8,9]. Moreover, Ozden and coworkers have described benefits on muscle strength and endurance when supervised core stability training is administered to patients after lumbar fusion [10]. In addition, the incorporation of cognitive-behavioral therapy into a physical exercise program has been demonstrated to decrease disability and fear of movement and improve the quality of life in these patients [10,11]. Improvements in psychosocial outcomes have also been reported to be positively associated with return to work in patients undergoing lumbar spine surgery [12].

However, the literature data reporting the effects of supervised rehabilitative interventions on psychosocial and participation-related outcomes have never been systematically analyzed, and a quantitative synthesis has never been carried out. Against this background, the aim of the current study was to systematically review the effects of supervised rehabilitation on quality of life, self-efficacy, fear-avoidance beliefs, catastrophizing, and return-to-work in patients after lumbar spine surgery.

## 2. Materials and Methods

This systematic review was conducted in accordance with the guidelines outlined in the Preferred Reporting Items for Systematic Reviews and Meta-Analysis (PRISMA) statement [13]. The protocol was registered on PROSPERO (CRD42024588982).

### 2.1. Data Source and Strategy

A systematic literature search was carried out electronically using the academic databases PubMed, EMBASE, CINAHL, PEDro, CENTRAL, and Google Scholar from inception until March 2024. The search strategy included terms related to lumbar spine surgery and rehabilitation, inclusive of exercise therapy and psychosocial interventions. The terms were searched as keywords and free words in the titles and abstracts, while no terms related to outcomes were used to increase search sensitivity. The extended version of the search strategy for each database is shown in Table S1.

### 2.2. Study Selection

Studies satisfying the following inclusion criteria were included: (1) randomized controlled trials (RCTs) including patients older than 18 years after discectomy, laminectomy, or fusion surgery; (2) postoperative supervised rehabilitation programs including exercise therapy, manual therapy, cognitive-behavioral approaches, or physical agents; (3) control group undergoing unsupervised rehabilitative interventions, education or no intervention; (4) at least one psychosocial outcome among fear-avoidance beliefs, pain catastrophizing and anxiety and depression or at least one participation-related outcome for quality of life and return to work assessed at 6 months or 1 year; and (5) studies written in English. Studies including physical agents alone, herbal medicine, homeopathy, or alternative medicines, such as experimental or control interventions, were excluded. Study selection procedures were carried out by two independent reviewers (E.D.M. and F.S.). The results of the literature search were imported into Rayyan software (https://www.rayyan.ai, accessed on 10 August 2024) for screening. First, duplicates were detected through the automatic facility of the software, manually crosschecked, and removed. Subsequently, titles and abstracts of retrieved records were manually screened to select potentially relevant studies. Finally, the full texts of records retained in the previous phase were screened to identify studies satisfying the eligibility criteria. A third reviewer (G.M.) facilitated the decision process in the case of disagreement.

### 2.3. Data Extraction and Risk of Bias Assessment

The following information was extracted from the included studies by two independent reviewers (D.D.L and F.S.): authors and publication year, participants’ features (e.g., sample size, demographic characteristics), preoperative clinical conditions and surgical procedure details, experimental and control interventions features (e.g., type and details of interventions, timing of treatment start and duration), and outcome measures related to domains of interest with the relative scores before and after the treatment. When multiple outcome measures were reported to assess the same construct, scores of the most valid and reliable outcome measures were extracted. A third reviewer double-checked data extraction procedures for accuracy (G.M.). Two independent reviewers (G.M., D.D.L.) assessed the methodological quality of the included studies using the revised Cochrane risk-of-bias tool for randomized trials (RoB-2) [14]. The RoB-2 provides an overall risk-of-bias judgment (low risk, some concerns, or high risk) based on a 5-domain tool, including (1) bias arising from the randomization process, (2) bias due to deviations from intended interventions, (3) bias due to missing outcome data, (4) bias in the measurement of the outcome, and (5) bias due to selective reporting of the results. In the case of patient-reported outcomes, the fourth domain of the RoB2 was not considered in the overall assessment [15]. A third reviewer facilitated the decision-making in the presence of disagreement during the rating process (D.C.).

### 2.4. Data Synthesis

Data synthesis was performed by two independent reviewers and carried out using the Review Manager 5.4 software (Version 5.4.1) from the Cochrane Library (F.S. and G.M, Oxford, UK). A third reviewer assessed data synthesis for accuracy (P.A.). Changes from pre-treatment to post-treatment or post-treatment data were extracted for intervention and control groups. Subsequently, the results of clinically homogeneous studies were pooled and categorized into “6 months after surgery” and “1 year after surgery” timepoint categories. Subgroup analyses for surgical procedures and the start of physiotherapy (before or 4 weeks after surgery) were also carried out. The magnitude of the effects was expressed as standardized mean differences (SMDs) with 95% confidence intervals (CI_95_) and was interpreted using Cohen’s *d* as small (lower than 0.2), moderate (between 0.2 and 0.8), and large (greater than 0.8) [16]. Heterogeneity was assessed through a visual inspection of the forest plots based on the I^2^ statistics. Specifically, values higher than 75% indicated considerable heterogeneity [17]. The Grading of Recommendations, Assessment, Development, and Evaluation (GRADE) approach was used to rate evidence certainty for each outcome of interest. The certainty of evidence was rated as high, moderate, low, or very low [18].

## 3. Results

**Figure 1:**
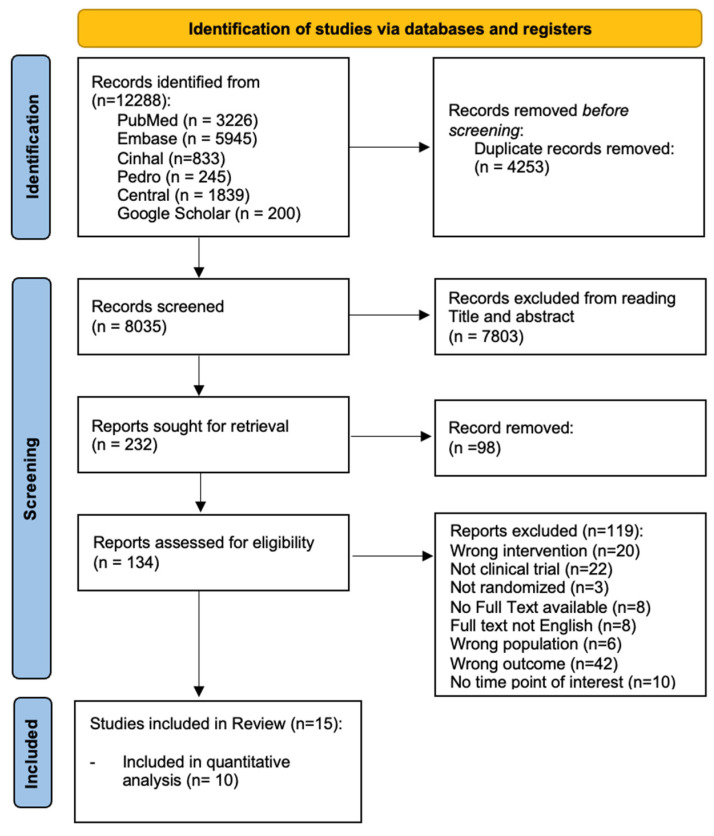
Flowchart of the study selection.

Twelve thousand two hundred and eight (12,208) potentially relevant records were identified throughout the literature search. After duplicate removal, 8035 records were screened for the title and abstract, and 7803 records were discarded. Finally, the full texts of 134 records were assessed for eligibility, and 15 studies were included in the current review (Figure 1). Specifically, 10 studies were included in the quantitative analysis, whereas 5 studies were analyzed only qualitatively due to their heterogeneity in outcome measures or non-normal data distribution.

### 3.1. Study Characteristics

The characteristics of the included studies are reported in Table 1. The current review included 1297 patients, of whom 595 were female. The sample size of the included studies ranged from 15 to 338 participants, with the mean age of participants ranging from 39 to 54 years. Twelve studies included patients after discectomy or laminectomy, while three studies included patients after fusion surgery (Table 1). All participants in the 15 studies underwent surgery for degenerative pathology.

The majority of studies delivered supervised interventions focused on exercise therapy, including strength, mobility, and muscle coordination exercises, aerobic training, and neuro-dynamic techniques. Two studies also administered cognitive-behavioral therapy in addition to exercise therapy [22,23]. When considering the control group, four studies delivered no intervention [21,30,32,33], six studies administered education on returning to work or ergonomic advice [19,20,24,26,27,31], and five studies delivered home-based exercise programs without supervision [22,23,25,28,29]. Finally, McGregor and co-workers delivered supervised exercise therapy with or without a leaflet as part of the experimental intervention (two groups) and surgeon’s advice or leaflet alone for the control intervention (two groups).

The timing of interventions ranged from hospital discharge to 3 months after surgery. Specifically, six studies started rehabilitation within 4 weeks after surgery, while nine studies started rehabilitative intervention after 4 weeks after surgery. The duration of interventions ranged from 1 to 3 months in 12 studies. Moreover, one study administered a 5-month intervention [31], while two studies administered a 1-year intervention [28,29].

When considering the outcomes, eight studies investigated quality of life. In particular, three studies reported results at 6 months [25,26,30], three studies reported findings at 1 year [22,24,28] and two studies included both timepoints [23,32]. Specifically, quality of life was assessed using EuroQoL5D-VAS, EuroQoL-5D, SF-12, RAND-36, and the Nottingham Health Profile. Eight studies investigated fear-avoidance belief using the Tampa Scale of Kinesiophobia (TSK) and the Fear Avoidance Belief Questionnaire (FAB-Q). In particular, this construct was assessed at both 6 months and 1 year in two studies and at 6 months only in two studies [25,26]. Self-efficacy was assessed by a single study at 6 months and 1 year using the Self-Efficacy Scale (SES). Catastrophizing was assessed by two studies using the Coping Strategy Questionnaire–Catastrophizing Scale (CSQ-CAT) [22,23]. Specifically, one study assessed catastrophizing at 6 months and 1 year, while one study investigated this construct at 1 year only. Anxiety and/or depression were investigated at 1 year in two studies using the Zung Depression Scale (ZDS) and Hospital Anxiety Depression Scale (HADS) [20,24]. Finally, three studies investigated the percentage of patients returning to work at 6 months [21,23,26], while a single study assessed the percentage of return to work both at 6 months and 1 year [33]. Finally, three studies assessed the length of the sick leave period [19,25,33].

### 3.2. Risk of Bias

**Figure 2:**
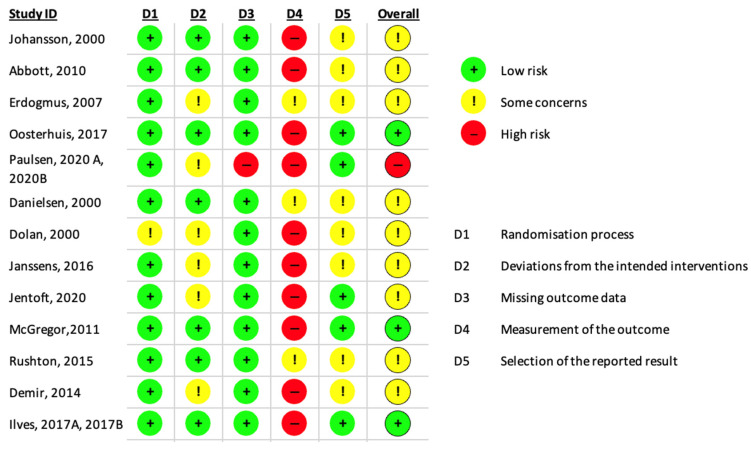
Risk of bias in included studies [19,20,21,22,23,24,25,26,27,28,29,30,31,32,33].

The risk of bias in the included studies is shown in Figure 2. The risk of bias was high in 27% of the studies; some concerns were found in 60% of studies, while the risk of bias was low in 13% of the studies included. In particular, one study had some concerns regarding the randomization process [20], two studies revealed missing outcome data [32,33], and eight studies revealed concerns related to the selective reporting of the results due to the lack of study protocol registration [19,20,21,22,23,25,26,27].

### 3.3. Quality of Life

**Figure 3:**
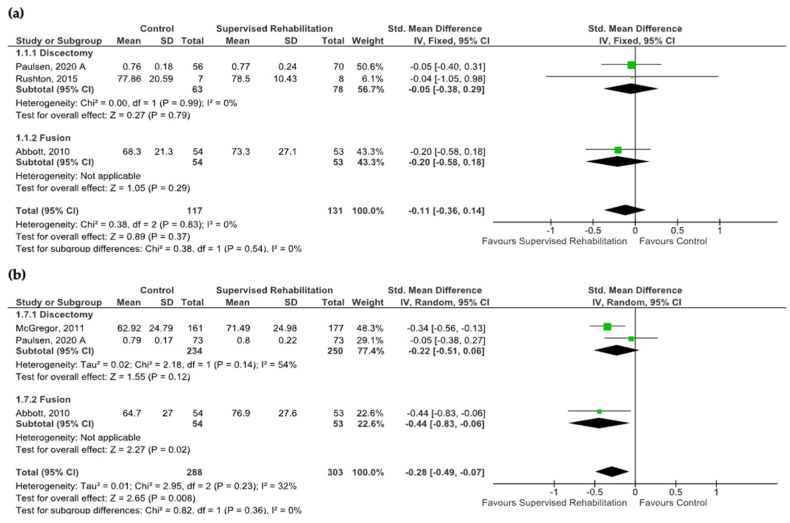
Pooled results on quality of life after lumbar spine surgery resulting from the comparison between supervised physiotherapy and the control group (unsupervised rehabilitative intervention, education, no treatment). Green squares represent the standardized mean difference (SMD) for each study, with their size proportional to the weight of each study. Diamonds represent the pooled SMD, with their width corresponding to the 95% CI. (**a**) Results 6 months after surgery and (**b**) 1 year after surgery [23,24,26,32].

Four studies were included in quantitative synthesis, while four studies were analyzed only qualitatively. When considering quantitative synthesis, no differences were found at 6 months, while supervised rehabilitation was found to be superior to the control intervention at 1 year with a moderate effect size (SMD: −0.28; CI_95_ from −0.49 to −0.07, I^2^ = 32%, *n* = 591) (very low evidence certainty) (Figure S1). No heterogeneity was detected at 6 months (I^2^ = 0%), whereas heterogeneity was moderate at 1 year (I^2^ = 32%). Subgroup analysis revealed that supervised physiotherapy improved quality of life in patients with fusion surgery at 1 year (SMD: −0.44; CI_95_ from −0.83 to −0.06, *n* = 107), while no effects were found in patients after discectomy. The results are shown in Figure 3. Subgroup analysis related to the timing of supervised rehabilitation started showing significant results in favor of supervised rehabilitation at 1 year (SMD: −0.44, CI_95_ from −0.83 to −0.06, *n* = 107), when rehabilitative interventions began within 4 weeks after surgery (Figure S2). When considering qualitative synthesis, no between-group differences were found, except for the study by Demir and co-workers revealing a better quality of life in favor of supervised rehabilitation at 6 months [25].

### 3.4. Fear-Avoidance Belief

**Figure 4:**
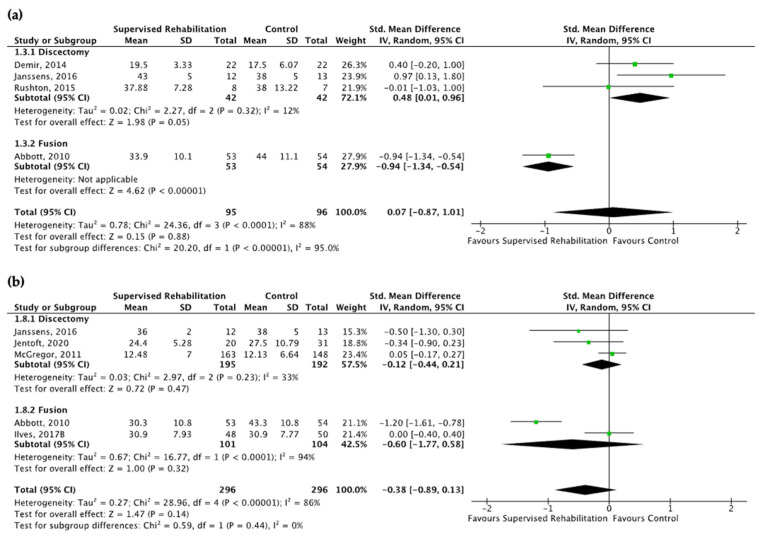
Pooled results for fear-avoidance belief after lumbar spine surgery resulting from the comparison between supervised physiotherapy and the control group (unsupervised rehabilitative intervention, education, no treatment). Green squares represent the standardized mean difference (SMD) for each study, with their size proportional to the weight of each study. Diamonds represent the pooled SMD, with their width corresponding to the 95% CI. (**a**) The results 6 months after surgery and (**b**) 1 year after surgery [23,24,25,26,27,29,31].

Supervised rehabilitation resulted in no superior results to the control intervention for fear-avoidance belief at 6 months and 1 year after surgery (very low evidence certainty) (Figure S1). High heterogeneity was found both at 6 months (I^2^ = 88%) and 1 year (I^2^ = 86%). The visual inspection of the forest plot suggested that heterogeneity was derived from the study of Abbott and co-workers. Subgroup analysis revealed a better fear of avoidance belief score in favor of the control group in patients after discectomy at 6 months (SMD: 0.48; CI_95_ from 0.01 to 0.96, I^2^ = 12%, *n* = 84), whereas the better fear of avoidance belief was found in favor of supervised rehabilitation in patients after spinal fusion at 6 months (SMD: −0.94, CI_95_ from −1.34 to −0.54, *n* = 107). The results are shown in Figure 4. Interestingly, significant results were found in favor of supervised rehabilitation at 1 year (SMD: −0.72, CI_95_ from −1.32 to −0.12, *n* = 183), when rehabilitative interventions began within 4 weeks after surgery (Figure S3). When considering these qualitative findings, Johannson and co-workers showed no differences between supervised rehabilitation starting within 4 weeks after surgery and the control group [22].

### 3.5. Catastrophizing and Self-Efficacy

**Figure 5:**
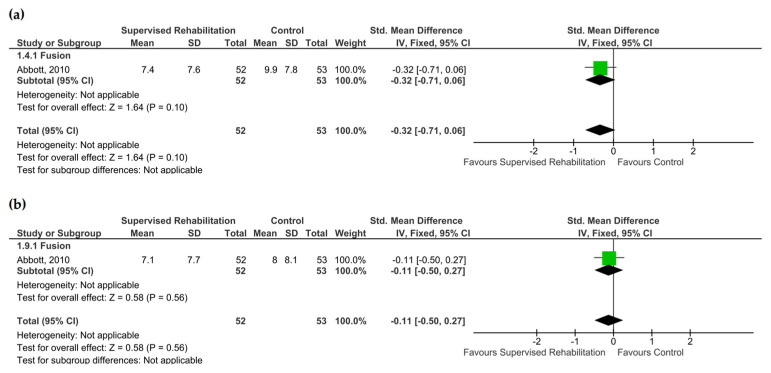
Pooled results for catastrophizing after lumbar spine surgery resulting from the comparison between supervised physiotherapy and the control group (unsupervised rehabilitative intervention, education, no treatment). Green squares represent the standardized mean difference (SMD) for each study, with their size proportional to the weight of each study. Diamonds represent the pooled SMD, with their width corresponding to the 95% CI. (**a**) The results 6 months after surgery and (**b**) 1 year after surgery [23].

**Figure 6:**
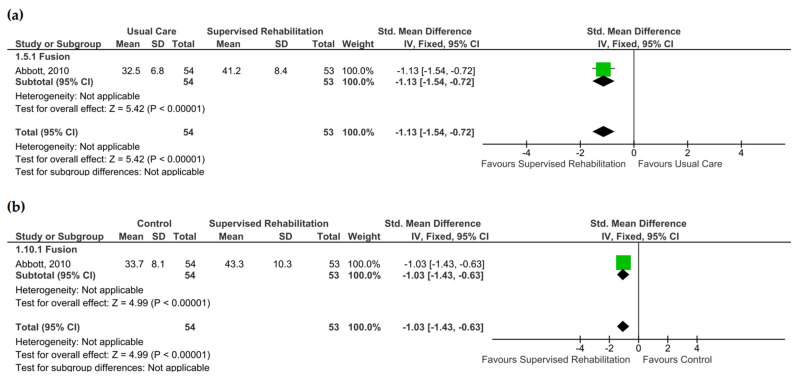
Pooled results for self-efficacy after lumbar spine surgery resulting from the comparison between supervised physiotherapy and the control group (unsupervised rehabilitative intervention, education, no treatment). Green squares represent the standardized mean difference (SMD) for each study, with their size proportional to the weight of each study. Diamonds represent the pooled SMD, with their width corresponding to the 95% CI. (**a**) The results 6 months after surgery and (**b**) 1 year after surgery [23].

Catastrophizing and self-efficacy were quantitatively evaluated by a single study, which revealed no superiority for supervised rehabilitation compared to the control intervention at 6 months and 1 year after surgery (low evidence certainty) [23] (Figure S1 and Figure 5). When considering the qualitative findings, Johansson and co-workers found no between-group differences in terms of catastrophizing [22]. Low evidence supported supervised rehabilitation to enhance self-efficacy at 6 months (SMD: −1.13; CI_95_ from −1.54 to −0.72, *n* = 107) and 1 year (SMD −1.03, CI_95_ from −1.43 to −0.63, *n* = 107) with a large effect size (Figure 6). All studies, including this outcome, implemented rehabilitative programs starting within 4 weeks after surgery (Figures S4 and S5).

### 3.6. Anxiety and Depression

**Figure 7:**
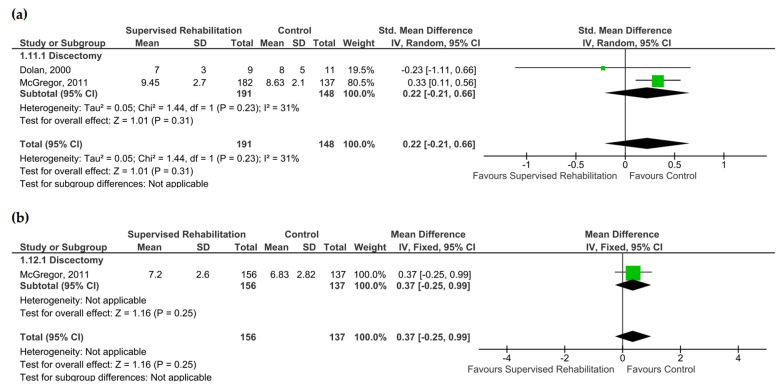
Pooled results for depression and anxiety after lumbar spine surgery resulting from the comparison between supervised physiotherapy and the control group (unsupervised rehabilitative intervention, education, no treatment). Green squares represent the standardized mean difference (SMD) for each study, with their size proportional to the weight of each study. Diamonds represent the pooled SMD, with their width corresponding to the 95% CI. (**a**) Depression 1 year after surgery and (**b**) anxiety 1 year after surgery [20,24].

No differences between supervised rehabilitation and the control group were found in terms of anxiety or depression (Figure 7). The certainty of evidence was low for anxiety and very low for depression (Figure S1). All studies, including this outcome, implemented rehabilitative programs starting 4 weeks post-surgery (Figure S6).

### 3.7. Return to Work

**Figure 8:**
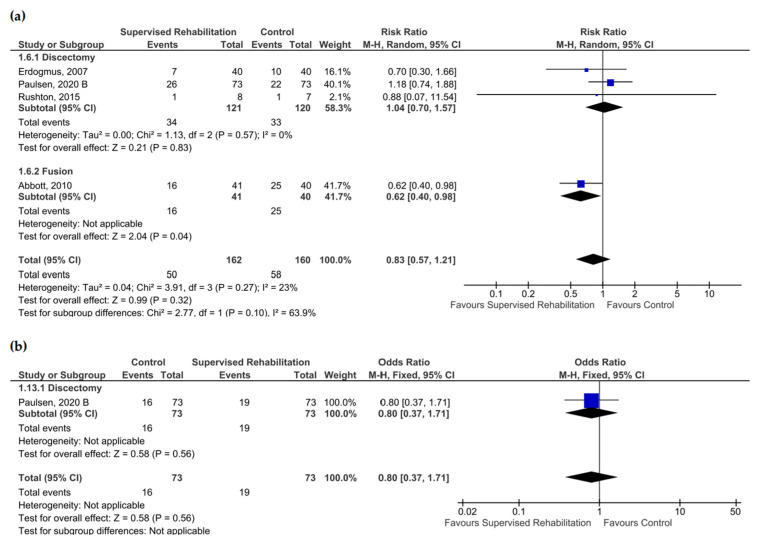
Pooled results for return to work after lumbar spine surgery comparing supervised physiotherapy with the control group (unsupervised rehabilitative intervention, education, no treatment). Blue squares represent the Odds Ratio (OR) for each study, with their size proportional to the weight of each study. Diamonds represent the pooled OR, with their width corresponding to the 95% CI. (**a**) The results 6 months after surgery and (**b**) 1 year after surgery [20,21,24,30].

No effects were found in favor of supervised rehabilitation at 6 months and 1 year after surgery (very low certainty of evidence). Sub-group analysis revealed that supervised rehabilitation improved the ability to return to work 6 months after lumbar fusion (OR: 0.38; CI_95_ from 0.16 to 0.94; *n* = 81) (Figure S1 and Figure 8). A significant improvement in return to work was detected in the supervised rehabilitation group (OR 0.64; CI_95_ from 0.43 to 0.95; *n* = 162) when rehabilitative interventions began within 4 weeks after surgery (Figure S7). When considering sick leave, qualitative analysis revealed no effects of supervised rehabilitation compared to the control group [19,25,33].

## 4. Discussion

The current systematic review revealed that supervised rehabilitation improved the quality of life at 1 year in patients after spinal surgery. Moreover, supervised rehabilitation enhanced self-efficacy at 6 months and 1 year in these patients. In addition, no effects of supervised rehabilitation were found in terms of quality of life at 6 months and fear-avoidance belief, catastrophizing, anxiety, depression, and return to work at 6 months and 1 year. However, the certainty of the evidence was low to very low, and further high-quality studies are needed.

Data from the literature have reported back and leg pain in patients undergoing lumbar spine surgery, leading to lower functional abilities and decreased quality of life [34]. In the current review, supervised rehabilitation has been reported to induce ameliorations in terms of quality of life at 1 year, whereas no effects were found at 6 months after surgery. Although the majority of included studies adopted exercise therapy as supervised rehabilitation, the aforementioned findings may derive from the fact that studies included in the 1-year timepoint compared supervised rehabilitation to no intervention [24,32]. On the other hand, studies included in the 6-month timepoint compared supervised rehabilitation with a home-based exercise protocol [26,32]. Therefore, it is reasonable to speculate that clinical heterogeneity in the control group protocols among the studies included at different timepoints might have influenced our findings. This interpretation is also consistent with the qualitative findings, which showed no differences in terms of quality of life between supervised rehabilitation and the control group [22,28]. In contrast, Demir and co-workers reported long-term benefits in favor of supervised rehabilitation compared to home-based exercises [25]. However, improvements were limited to two out of six domains of the Nottingham Health Profile, and the data of the questionnaire score were not reported in the study [25]. In light of these findings, low evidence of certainty and a small number of studies, additional studies are needed to assess the effects of a supervised rehabilitative program on quality of life in patients after lumbar spine surgery.

The current systematic review also supported supervised rehabilitation to enhance self-efficacy at 6 months and 1 year after lumbar spine surgery. These findings derive from the study of Abbott and co-workers, which combined exercise therapy with cognitive-behavioral therapy and began rehabilitation at hospital discharge. Self-efficacy is defined as the confidence of a subject’s ability to perform a task, and these findings highlighted the importance of the biopsychosocial approach in the postoperative rehabilitation pathway of patients after spinal surgery [35]. Individual rehabilitation projects tailored to patients’ characteristics and rehabilitation cycles carried out by a multidisciplinary team deserve to be considered to improve psychosocial outcomes in these patients [36]. In fact, in addition to physical exercises, interventions targeted to personal and contextual factors of the International Classification of Functioning (ICF) model have been recommended to improve psychosocial and participation-related outcomes in patients undergoing lumbar spine surgery [11]. Similarly, tools for assessing the effects of rehabilitative interventions on disability and health according to the ICF principles and independent of diagnosis (e.g., World Health Organization Disability Assessment Schedule 2.0) deserve to be taken into account [37]. However, the certainty of evidence regarding the effects of supervised rehabilitation on self-efficacy is low, and the findings derive from a single study. Therefore, caution is needed in interpreting the current results.

This systematic review found no superiority when using supervised rehabilitation on fear-avoidance belief. However, our subgroup analysis found a large effect size at 6 months in favor of supervised rehabilitation in patients undergoing fusion surgery, while no effect related to the time at which rehabilitation started was found. Such an effect was driven by the study of Abbott and co-workers, where the combination of exercise and cognitive-behavioral therapies might have contributed to improving patients’ maladaptive behaviors [23]. In fact, cognitive-behavioral techniques are aimed at teaching coping mechanisms and providing support to subjects experiencing feelings of distress [38]. When considering fear-avoidance belief at 1 year in patients with fusion surgery, no effects were found. The subgroup analysis included two studies: one adopting a protocol including exercise plus cognitive-behavioral therapy [23], and one adopting a protocol focused on exercises aimed at enhancing muscle strength, coordination, and promoting physical activity [29]. In this scenario, it is reasonable to speculate that clinical heterogeneity deriving from the contents of supervised rehabilitative approaches might have influenced the findings in patients after spinal fusion at 1 year. Additionally, although the timing of starting supervised rehabilitation was considered a potential determinant of efficacy, the findings seem to be driven by the results of Abbott and co-workers, who implemented a cognitive-behavioral approach and therapeutic exercises into a supervised rehabilitation program from hospital discharge. No supervised rehabilitation effects were found on catastrophizing at 6 months and 1 year. Catastrophizing represents a common psychosocial factor affecting the postoperative recovery of patients undergoing spinal surgery [39,40,41]. Although our findings are based on two studies starting supervised rehabilitation within 4 weeks after surgery, the current results are in contrast with some data in the literature [11]. In fact, Monticone and co-workers reported that a supervised rehabilitation program, including strategies for the management of catastrophizing, was superior to exercise therapy alone in patients after lumbar fusion. However, the treatment posology of approaches that addressed catastrophizing in our included studies was substantially different from the posology adopted by Monticone and co-workers. In particular, Monticone and co-workers administered two weekly sessions for 4 weeks to patients, whereas studies included in the current review delivered only three sessions [23], or a single weekly session [22]. Similarly, the current review suggested that supervised rehabilitation revealed no effects on anxiety and depression assessed at 1 year after surgery. However, the current findings are based on two studies assessing anxiety and a single study investigating depression. Therefore, although the incidence of anxiety and depression onset in patients after spinal surgery has been reported to be 11% and 6%, respectively, additional studies are needed to assess the effects of supervised rehabilitation on anxiety and depression in patients after spinal surgery [42].

Supervised rehabilitation revealed no effects on returning to work in patients after spinal surgery at 6 months and 1 year. Previous studies have shown that the majority of patients undergoing discectomy or lumbar fusion returned to work within 3 months [43], suggesting that a shorter timepoint deserves to be considered in these patients. Interestingly, the current review found that the sub-group of patients undergoing lumbar fusion seemed to be more likely to return to work within 6 months when they underwent a supervised postoperative rehabilitative program. The aforementioned results are derived from a single study in which the control group received a home-based exercise program only with no cognitive-behavioral approach [23]. Therefore, the contents of the rehabilitative interventions may play a pivotal role in determining the current findings, and the trajectories of functional recovery were described as similar in patients undergoing lumbar discectomy and fusion [44,45]. In addition, a better return to work rate induced by a supervised rehabilitation program was found when the intervention began within 4 weeks after surgery [21,23]. However, when considering the adoption of heterogeneous rehabilitative approaches in studies delivering early rehabilitation, future studies are needed to clarify the role of timing of rehabilitation initiation on return to work in patients after spinal surgery [21,23].

## 5. Limitations

Some limitations of the current systematic review deserve to be highlighted. First, the sample size was relatively small in most of the studies, leading to a possible decrease in the precision of pooled effect estimates for some outcomes. Second, the results of the current review are partially based on qualitative data synthesis for some outcomes, including a lack of reported data in some included studies. Third, included studies had wide variability in terms of the contents of experimental and control interventions, the duration (from 4 weeks to 3 months), and the timing of treatment initiation (from a few days after surgery to 6–8 weeks after surgery), increasing the heterogeneity. Finally, only a limited number of studies assessed all outcomes, and some conclusions were drawn on data deriving from a single study, leading to a low or very low certainty of evidence.

## 6. Conclusions

Supervised rehabilitation improved self-efficacy at 6 months and enhanced quality of life and self-efficacy at 1 year after spinal surgery, suggesting the opportunity to adopt supervised programs into postoperative rehabilitative protocols for these patients. No effects of supervised rehabilitation were found in terms of quality of life at 6 months and fear-avoidance belief, catastrophizing, anxiety, depression, and return to work at 6 months and 1 year. The certainty of the evidence is still limited, and further high-quality studies are needed.

## Figures and Tables

**Table 1:** Characteristics of included studies.

Author, Year [Reference]	No. Patients and Gender	Experimental Group	Control Group	Timing of Treatment Start	Outcome Measures and Timepoint
Danielsen et al., 2000 [19]	63 patients after discectomy M/F: 41/66	Supervised strength exercises for lumbar muscles, core muscles, and lower limb muscles for 8 weeks.	Information on the self-management of the first 2 months postoperative pathway and home-based exercise training program.	4 weeks after surgery.	Return to work (sick leave period).
Dolan et al., 2000 [20]	20 patients after discectomy M/F: 17/3	Supervised aerobic exercises, stretching, and lumbar extension exercises for 8 weeks.	Usual postoperative care and information about exercises and return to work management.	6 weeks after surgery.	1 year: - Zung Depression Scale.
Erdogmus et al., 2007 [21]	99 patients after discectomy M/F: 67/32	Supervised strength training and mobilization, stretching and stabilization exercises, ergonomic activities, and work-return advice for 12 weeks.	No treatment.	1 week after surgery.	6 months: - Return to work (Yes/No).
Johansson et al., 2009 [22]	59 patients after discectomy M/F: 35/24	Supervised exercises with positive reinforcement of healthy behaviors for 8 weeks.	Home-based unsupervised exercises with recommendations on gradual increases in exercise intensity.	2 weeks after surgery.	1 year: - EQ-5D VAS - TSK - CSQ-CAT.
Abbott et al., 2010 [23]	107 patients after spinal fusion M/F: 41/66	Supervised sessions with education for modifying maladaptive pain cognition, behavior, and performance of motor control exercise, plus home-based exercises for 12 weeks.	Home-based exercise program.	Hospital discharge (not specified).	6 months: - EQ-5D - TSK - SES - CSQ-CAT. 1 year: - EQ-5D - TSK - SES - CSQ-CAT.
McGregor et al., 2011 [24]	338 patients after discectomy M/F: 159/179	Group 1: supervised stretching, strength training, aerobic training, endurance, and ergonomic exercises for 6 weeks. Group 2: performance of the same exercises as Group 1 for 6 weeks plus informational booklet.	Group 1: information booklet. Group 2: Usual care according to advice delivered by the surgeon.	6–8 weeks after surgery.	1 year: - EQ-5D VAS - FAB-Q - HADS.
Demir et al., 2014 [25]	44 patients after discectomy M/F: 24/20	Supervised dynamic lumbar stabilization exercises for 4 weeks.	Home-based exercise program, including stretching, pelvic tilt mobilization, flexion, and extension exercises for 4 weeks.	4 weeks after surgery.	6 months: - Nottingham Health Profile - FAB-Q - Return to work (sick leave period).
Rushton et al., 2015 [26]	59 patients after discectomy M/F: 28/31	Supervised treatment including education, advice, mobility, and core stability exercises for 8 weeks.	Leaflet regarding anatomical and surgical information, advice on progressive exercises execution, and answers to frequently asked questions.	4 weeks after surgery.	6 months: - EQ-5D - TSK - Return to work (Yes/No).
Janssens et al., 2016 [27]	25 patients after discectomy M/F: 11/14	Supervised sessions focused on correction of sitting posture, deep abdominal muscles exercises, neurodynamic exercises, and segmental thoracolumbar mobilization for 2 weeks.	Ergonomic advice and the “*stay active*” advice.	2 weeks after surgery.	6 months: - TSK 1 year: - TSK - Return to work (sick leave period).
Ilves et al., 2017 (A) [28] and Ilves et al., 2017 (B) [29]	98 patients after spinal fusion M/F: 72/26	Supervised sessions with a physiotherapist and home-based program focused on back-specific exercises and aerobic exercises for 52 weeks.	Instructions for standard home-based exercises.	12 weeks after surgery.	1 year: - RAND-36 (A) - TSK (B).
Oosterhuis et al., 2017 [30]	169 patients after discectomy M/F: 71/98	Supervised usual postoperative exercises for 6–8 weeks.	No postoperative rehabilitation.	3 days after surgery.	6 months - SF-12.
Jentoft et al., 2020 [31]	70 patients after discectomy M/F: 44/26	Supervised stretching and muscle coordination exercises, strength training for 18–20 weeks, and education at discharge.	Educational session at discharge on spine anatomy, pain physiology, surgery intervention, and suggested postoperative activities.	At discharge.	1 year: - TKS.
Paulsen et al., 2020 (A) [32] and Paulsen et al., 2020 (B) [33]	146 patients after discectomy M/F: 92/54	Supervised spinal stability exercises for 6–12 weeks.	No postoperative rehabilitation.	4–6 weeks after surgery.	6 months: - EQ-5D (A) - Return to work (Yes/No) (B). 1 year: - EQ-5D (A) - Return to work (Yes/No) (B).

Abbreviations: M: male; F: female; EQ-5D: EuroQOL-5 Dimension; VAS: visual analog scale; TSK: Tampa Scale of Kinesiophobia; CSQ-CAT: Coping Strategies Questionnaire–Catastrophizing Scale; SES: Self-Efficacy Scale; FAB-Q: Fear Avoidance Belief Questionnaire; HADS: Hospital Anxiety and Depression Scale; SF-12: Short-Form Health Survey.

## Data Availability

The data that support the findings of this study are available from the corresponding author upon reasonable request.

## Supplementary Materials

The following supporting information can be downloaded at https://www.mdpi.com/article/10.3390/jcm13237246/s1. Table S1: Search strategy; Figure S1: SoF table; Figures S2–S7: Subgroup analysis results on the timing of supervised rehabilitation start.
