# Supplementary material for: Effects of Supervised Rehabilitation on Psychosocial and Participation-Related Outcomes After Lumbar Spine Surgery: A Systematic Review and Meta-Analysis
Source: J Clin Med. 2024 Nov 28;13(23):7246. doi: 10.3390/jcm13237246 (PMC11642663; doi:10.3390/jcm13237246)
Supplement: Supplementary file 1 [file jcm-13-07246-s001.zip › jcm-3304088-supplementary.pdf]

Table S1. Search strategy for each database**.**

Date: 25, March 2024.

MEDLINE (via Pubmed)
(("lumbosacral region"[MeSH Terms] OR lumbosacral[Title/Abstract] OR "lumbosacral region"[Title/Abstract] OR "lumbar"[Title/Abstract] OR "Lumbar Vertebrae"[MeSH Terms] OR "low back"[Title/Abstract]) AND ("diskectomy"[MeSH Terms] OR "diskectom*"[Title/Abstract] OR "discectom*"[Title/Abstract] OR "decompression"[MeSH Terms] OR "decompress*"[Title/Abstract] OR "laminectomy"[MeSH Terms] OR "laminectom*"[Title/Abstract] OR "fusion"[Title/Abstract] OR "surger*"[Title/Abstract] OR "general surgery"[MeSH Terms])) AND ("exercise"[MeSH Terms] OR "exercise*"[Title/Abstract] OR "exercise therapy"[MeSH Terms] OR "exercise therapy"[Title/Abstract] OR "physical therapy modalities"[MeSH Terms] OR "physiotherapy"[Title/Abstract] OR "rehabilitation"[MeSH Terms] OR "rehabilitati*"[Title/Abstract] OR "manual therap*"[Title/Abstract] OR "Musculoskeletal Manipulations"[Mesh] OR "training program*"[Title/Abstract] OR "aquatic therap*"[Title/Abstract] OR "psychosocial intervention*"[Title/Abstract] OR "cognitive behavioral therap*"[Title/Abstract] OR "Cognitive Behavioral Therapy"[Mesh] OR "neural mobilization*"[Title/Abstract] OR "nerve stimulation*"[Title/Abstract] OR "Transcutaneous Electric Nerve Stimulation"[Mesh] OR "Transcutaneous Electric Nerve Stimulation"[Title/Abstract] OR "yoga"[Title/Abstract] OR "Yoga"[Mesh] OR "pilates"[Title/Abstract] OR "tai chi"[Title/Abstract] OR "core stability"[Title/Abstract] OR "hydrotherapy*"[Title/Abstract] OR "Hydrotherapy"[Mesh] OR "aerobic training"[Title/Abstract] OR "resistance training"[Title/Abstract] OR "Resistance Training"[Mesh] OR "strength training"[Title/Abstract] OR "endurance training"[Title/Abstract] OR "Endurance Training"[Mesh]) **Results**: 3226<br>1. ("lumbosacral region"[MeSH Terms] OR lumbosacral[Title/Abstract] OR "lumbosacral region"[Title/Abstract] OR "lumbar"[Title/Abstract] OR "Lumbar Vertebrae"[MeSH Terms] OR "low back"[Title/Abstract]) AND ("diskectomy"[MeSH Terms] OR "diskectom*"[Title/Abstract] OR "discectom*"[Title/Abstract] OR "decompression"[MeSH Terms] OR "decompress*"[Title/Abstract] OR "laminectomy"[MeSH Terms] OR "laminectom*"[Title/Abstract] OR "fusion"[Title/Abstract] OR "surger*"[Title/Abstract] OR "general surgery"[MeSH Terms]) Results: 50894<br>2. ("exercise"[MeSH Terms] OR "exercise*"[Title/Abstract] OR "exercise therapy"[MeSH Terms] OR "exercise therapy"[Title/Abstract] OR "physical therapy modalities"[MeSH Terms] OR "physiotherapy"[Title/Abstract] OR "rehabilitation"[MeSH Terms] OR "rehabilitati*"[Title/Abstract] OR "manual therap*"[Title/Abstract] OR "Musculoskeletal Manipulations"[Mesh] OR "training program*"[Title/Abstract] OR "aquatic therap*"[Title/Abstract] OR "psychosocial intervention*"[Title/Abstract] OR "cognitive behavioral therap*"[Title/Abstract] OR "Cognitive Behavioral Therapy"[Mesh] OR "neural mobilization*"[Title/Abstract] OR "nerve stimulation*"[Title/Abstract] OR "Transcutaneous Electric Nerve Stimulation"[Mesh] OR "Transcutaneous Electric Nerve Stimulation"[Title/Abstract] OR "yoga"[Title/Abstract] OR "Yoga"[Mesh] OR "pilates"[Title/Abstract] OR "tai chi"[Title/Abstract] OR "core stability"[Title/Abstract] OR "hydrotherapy*"[Title/Abstract] OR "Hydrotherapy"[Mesh] OR "aerobic training"[Title/Abstract] OR "resistance training"[Title/Abstract] OR "Resistance Training"[Mesh] OR "strength training"[Title/Abstract] OR "endurance training"[Title/Abstract] OR "Endurance Training"[Mesh]) Results: 1 067 445<br>3. 1 AND 2 Results: 3226
**EMBASE**
('lumbosacral region'/exp OR 'lumbosacral':ti,ab,kw OR 'lumbosacral region':ti,ab,kw OR 'lumbar':ti,ab,kw OR 'lumbar vertebra'/exp OR 'low back':ti,ab,kw) AND ('discectomy'/exp OR 'diskectom*':ti,ab,kw OR 'discectom*':ti,ab,kw OR 'decompression'/exp OR 'decompress*':ti,ab,kw OR 'laminectomy'/exp OR 'laminectom*':ti,ab,kw OR 'fusion':ti,ab,kw OR 'surger*':ti,ab,kw OR 'general surgery'/exp) AND ('exercise'/exp OR 'exercise*':ti,ab,kw OR 'kinesiotherapy'/exp OR 'exercise therapy':ti,ab,kw OR 'physiotherapy'/exp OR 'physiotherapy':ti,ab,kw OR 'rehabilitation'/exp OR 'rehabilitati*':ti,ab,kw OR 'manual therap*':ti,ab,kw OR 'musculoskeletal manipulation'/exp OR 'training program*':ti,ab,kw OR 'exercise*':ti,ab,kw OR 'aquatic therap*':ti,ab,kw OR 'psychosocial intervention*':ti,ab,kw OR 'cognitive behavioral therap*':ti,ab,kw OR 'cognitive behavioral therapy'/exp OR 'neural mobilization*':ti,ab,kw OR 'nerve stimulation*':ti,ab,kw OR 'transcutaneous electrical nerve stimulation'/exp OR 'transcutaneous electric nerve stimulation':ti,ab,kw OR 'yoga':ti,ab,kw OR 'yoga'/exp OR 'pilates':ti,ab,kw OR 'tai chi':ti,ab,kw OR 'core stability':ti,ab,kw OR 'hydrotherapy*':ti,ab,kw OR 'hydrotherapy'/exp OR 'aerobic training':ti,ab,kw OR 'resistance training':ti,ab,kw OR 'resistance training'/exp OR 'strength training':ti,ab,kw OR 'endurance training':ti,ab,kw OR 'endurance training'/exp) **Results**: 5945<br>1. ('lumbosacral region'/exp OR 'lumbosacral':ti,ab,kw OR 'lumbosacral region':ti,ab,kw OR 'lumbar':ti,ab,kw OR 'lumbar vertebra'/exp OR 'low back':ti,ab,kw) AND ('discectomy'/exp OR 'diskectom*':ti,ab,kw OR 'discectom*':ti,ab,kw OR 'decompression'/exp OR 'decompress*':ti,ab,kw OR 'laminectomy'/exp OR 'laminectom*':ti,ab,kw OR 'fusion':ti,ab,kw OR 'surger*':ti,ab,kw OR 'general surgery'/exp) Results: 65889<br>2. ('exercise'/exp OR 'exercise*':ti,ab,kw OR 'kinesiotherapy'/exp OR 'exercise therapy':ti,ab,kw OR 'physiotherapy'/exp OR 'physiotherapy':ti,ab,kw OR 'rehabilitation'/exp OR 'rehabilitati*':ti,ab,kw OR 'manual therap*':ti,ab,kw OR 'musculoskeletal

manipulation'/exp OR 'training program*':ti,ab,kw OR 'aquatic therap*':ti,ab,kw OR 'psychosocial intervention*':ti,ab,kw OR 'cognitive behavioral therap*':ti,ab,kw OR 'cognitive behavioral therapy'/exp OR 'neural mobilization*':ti,ab,kw OR 'nerve stimulation*':ti,ab,kw OR 'transcutaneous electrical nerve stimulation'/exp OR 'transcutaneous electric nerve stimulation':ti,ab,kw OR 'yoga':ti,ab,kw OR 'yoga'/exp OR 'pilates':ti,ab,kw OR 'tai chi':ti,ab,kw OR 'core stability':ti,ab,kw OR 'hydrotherapy*':ti,ab,kw OR 'hydrotherapy'/exp OR 'aerobic training':ti,ab,kw OR 'resistance training':ti,ab,kw OR 'resistance training'/exp OR 'strength training':ti,ab,kw OR 'endurance training':ti,ab,kw OR 'endurance training'/exp) Results: 1 512 950

3. 1 AND 2 Results: 5945

CINAHL (via EBSCOhost)

AB ((lumbosacral region) OR lumbar OR ("low back")) AND (diskectomy OR decompression OR laminectomy OR fusion OR surgery)) AND AB (exercise OR ("physical therapy") OR physiotherapy OR rehabilitation OR ("manual therapy") OR ("aquatic therapy") OR ("psychosocial intervention") OR ("cognitive behavioral therapy") OR ("neural mobilization") OR ("nerve stimulation") OR ("neural stimulation") OR ("transcutaneous electric nerve stimulation") OR yoga OR pilates OR ("tai chi") OR ("core stability") OR hydrotherapy OR ("aerobic training") OR ("resistance training") OR ("strength training") OR ("endurance training")) **Results**: 833

1. AB ((lumbosacral region) OR lumbar OR ("low back")) AND (diskectomy OR decompression OR laminectomy OR fusion OR surgery)) Results: 10572
2. AB(exercise OR ("physical therapy") OR physiotherapy OR rehabilitation OR ("manual therapy") OR ("aquatic therapy") OR ("psychosocial intervention") OR ("cognitive behavioral therapy") OR ("neural mobilization") OR ("nerve stimulation") OR ("neural stimulation") OR ("transcutaneous electric nerve stimulation") OR yoga OR pilates OR ("tai chi") OR ("core stability") OR hydrotherapy OR ("aerobic training") OR ("resistance training") OR ("strength training") OR ("endurance training")) Results: 213512
3. 1 AND 2 Results 833

PEDro

Simple search: (The search was conducted as suggested by PEDro: "Reduce the number of search terms (many searches are best performed by using only one search term in one of the search fields)." **Results:** 245

1. Lumbar. Results: 2458
2. Lumbar, surgery. Results: 1063
3. Lumbar, surgery, rehabilitation. Results: 87
4. Lumbar, surgery, exercise. Results: 158
5. Line 3 and 4 together resulted: 245

CENTRAL

TITLE-ABS-KEY ((lumbosacral AND region) OR lumbar OR (low AND back)) AND (diskectomy OR decompression OR laminectomy OR fusion OR surgery) AND (exercise OR (physical AND therapy) OR physiotherapy OR rehabilitation OR (manual AND therapy) OR (aquatic AND therapy) OR (psychosocial AND interventions) OR (cognitive AND behavioral AND therapy) OR (neural AND mobilization) OR (nerve AND stimulation) OR (neural AND stimulation) OR (transcutaneous AND electric AND nerve AND stimulation) OR yoga OR pilates OR (tai AND chi) OR (core AND stability) OR hydrotherapy OR (aerobic AND training) OR (resistance AND training) OR (strength AND training) OR (endurance AND training) **Results**: 1839 (trials)

1. (lumbosacral AND region) OR lumbar OR (low AND back)) AND (diskectomy OR decompression OR laminectomy OR fusion OR surgery) Results: 8959
2. (exercise OR (physical AND therapy) OR physiotherapy OR rehabilitation OR (manual AND therapy) OR (aquatic AND therapy) OR (psychosocial AND interventions) OR (cognitive AND behavioral AND therapy) OR (neural AND mobilization) OR (nerve AND stimulation) OR (neural AND stimulation) OR (transcutaneous AND electric AND nerve AND stimulation) OR yoga OR pilates OR (tai AND chi) OR (core AND stability) OR hydrotherapy OR (aerobic AND training) OR (resistance AND training) OR (strength AND training) OR (endurance AND training) Results: 290610
3. 1 AND 2. Results: 1898 of which 1839 were trials

Google Scholar

((lumbosacral AND region) OR lumbar OR (low AND back)) AND (diskectomy OR decompression OR laminectomy OR fusion OR surgery)) AND (exercise OR (physical AND therapy) OR physiotherapy OR rehabilitation OR (manual AND therapy) OR (aquatic AND therapy) OR (psychosocial AND interventions) OR (cognitive AND behavioral AND therapy) OR (neural AND mobilization) OR (nerve AND stimulation)

OR (neural AND stimulation) OR (transcutaneous AND electric AND nerve AND stimulation) OR yoga OR pilates OR (tai AND chi) OR (core AND stability) OR hydrotherapy OR (aerobic AND training) OR (resistance AND training) OR (strength AND training) OR (endurance AND training) **Results:** 200

1. ((lumbosacral AND region) OR lumbar OR (low AND back)) AND (diskectomy OR decompression OR laminectomy OR fusion OR surgery)) Results: 85500
2. (exercise OR (physical AND therapy) OR physiotherapy OR rehabilitation OR (manual AND therapy) OR (aquatic AND therapy) OR (psychosocial AND interventions) OR (cognitive AND behavioral AND therapy) OR (neural AND mobilization) OR (nerve AND stimulation) OR (neural AND stimulation) OR (transcutaneous AND electric AND nerve AND stimulation) OR yoga OR pilates OR (tai AND chi) OR (core AND stability) OR hydrotherapy OR (aerobic AND training) OR (resistance AND training) OR (strength AND training) OR (endurance AND training) Results: 20200
3. 1 AND 2 Results 5150

Results: As suggested by Bramer et Al. (2017) first 200 revelevant reference were considered

TOTAL results: 12288

Results after duplicated removed: 4254 (with Rayyan Detect Duplicates tool)

Figure S1 GRADE Summary of Findings table. Supervised physiotherapy compared to control group (unsupervised rehabilitative intervention, education, no treatment) in patients 6 months and 1 year after after lumbar spine surgery.

Certainty assessment							№ of patients		Effect		Certainty	Importance
№ of studies	Study design	Risk of bias	Inconsistency	Indirectness	Imprecision	Other considerations	PT	Control	Relative (95% CI)	Absolute (95% CI)		
**Quality of life 6 months**												
3	randomised trials	serious	not serious	serious	serious[a]	none	131	117	-	SMD **0.11 SD lower** (0.36 lower to 0.14 higher)	⊕◯◯◯ Very low[a]	
**Fear Avoidance Belief 6 months**												
4	randomised trials	not serious	very serious[b]	serious	serious[a]	none	95	96	-	SMD **0.07 SD higher** (0.87 lower to 1.01 higher)	⊕◯◯◯ Very low[a,b]	
**Catastrophizing 6 months**												
1	randomised trials	not serious	not serious	serious	serious[a]	none	52	53	-	SMD **0.32 SD lower** (0.71 lower to 0.06 higher)	⊕⊕◯◯ Low[a]	
**Self-Efficacy 6 months**												
1	randomised trials	not serious	not serious	serious	serious[a]	none	54	53	-	SMD **1.13 SD lower** (1.54 lower to 0.72 lower)	⊕⊕◯◯ Low[a]	
**Return to work 6 months**												
4	randomised trials	serious	not serious	serious	serious[a]	none	50/162 (30.9%)	58/160 (36.3%)	**RR 0.83** (0.57 to 1.21)	**62 fewer per 1.000** (from 156 fewer to 76 more)	⊕◯◯◯ Very low[a]	
**Quality of life 1 year**												
3	randomised trials	serious	not serious	serious	serious[a]	none	303	288	-	SMD **0.28 lower** (0.49 lower to 0.07 lower)	⊕◯◯◯ Very low[a]	
**Fear Avoidance Belief 1 Year**												
5	randomised trials	not serious	very serious[b]	serious	serious[a]	none	296	296	-	SMD **0.38 SD lower** (0.89 lower to 0.13 higher)	⊕◯◯◯ Very low[a,b]	
**Catastrophizing 1 year**												
1	randomised trials	not serious	not serious	serious	serious[a]	none	52	53	-	SMD **0.11 SD lower** (0.5 lower to 0.27 higher)	⊕⊕◯◯ Low[a]	
**Self-Efficacy 1 year**												
1	randomised trials	not serious	not serious	serious	serious[a]	none	53	54	-	SMD **1.03 SD lower** (1.43 lower to 0.63 lower)	⊕⊕◯◯ Low[a]	
**Depression 1 year**												
1	randomised trials	not serious	not serious	serious	serious[a]	none	156	137	-	SMD **0.37 SD higher** (0.25 lower to 0.99 higher)	⊕⊕◯◯ Low[a]	
**Return to work 1 year**												
1	randomised trials	very serious	not serious	serious	serious[a]	none	19/73 (26.0%)	3/57 (5.3%)	**RR 0.80** (0.37 to 1.71)	**11 fewer per 1.000** (from 33 fewer to 37 more)	⊕◯◯◯ Very low[a]	

CI: confidence interval; **RR:** risk ratio; **SMD:** standardised mean difference

Explanations

a. Downgraded by one level because of small population
b. High heterogeneity
c. In meta-analysis there are opposite effects among studies

(a)

Study or Subgroup	Control Mean	Control SD	Control Total	Supervised Rehabilitation Mean	Supervised Rehabilitation SD	Supervised Rehabilitation Total	Weight	Std. Mean Difference IV, Fixed, 95% CI
**1.1.1 Early approach**								
Abbott, 2010	68.3	21.3	54	73.3	27.1	53	43.3%	−0.20 [−0.58, 0.18]
**Subtotal (95% CI)**			**54**			**53**	**43.3%**	**−0.20 [−0.58, 0.18]**
Heterogeneity: Not applicable								
Test for overall effect: Z = 1.05 (P = 0.29)								
**1.1.2 Late approach**								
Paulsen, 2020 A	0.76	0.18	56	0.77	0.24	70	50.6%	−0.05 [−0.40, 0.31]
Rushton, 2015	77.86	20.59	7	78.5	10.43	8	6.1%	−0.04 [−1.05, 0.98]
**Subtotal (95% CI)**			**63**			**78**	**56.7%**	**−0.05 [−0.38, 0.29]**
Heterogeneity: Chi^2 = 0.00, df = 1 (P = 0.99); I^2 = 0%								
Test for overall effect: Z = 0.27 (P = 0.79)								
**Total (95% CI)**			**117**			**131**	**100.0%**	**−0.11 [−0.36, 0.14]**
Heterogeneity: Chi^2 = 0.38, df = 2 (P = 0.83); I^2 = 0%								
Test for overall effect: Z = 0.89 (P = 0.37)								
Test for subgroup differences: Chi^2 = 0.38, df = 1 (P = 0.54), I^2 = 0%								

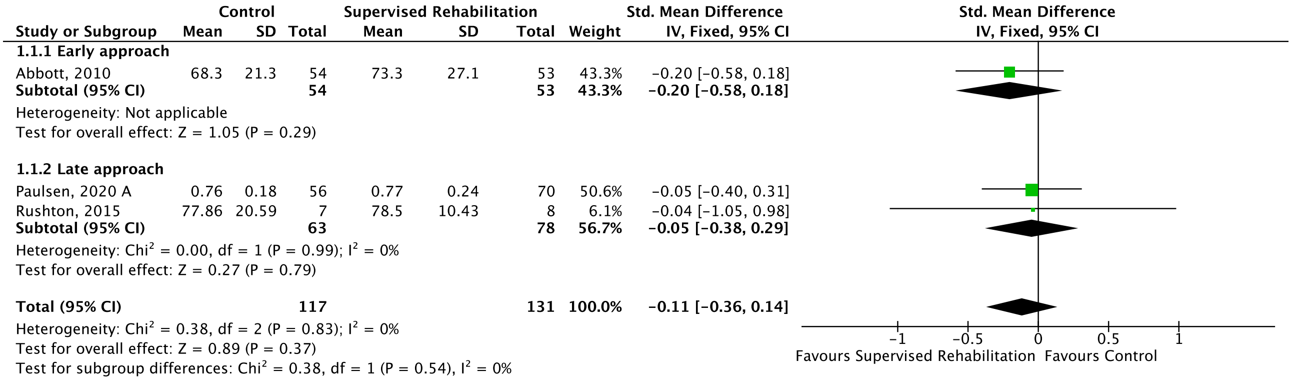

(b)

Study or Subgroup	Control Mean	Control SD	Control Total	Supervised Rehabilitation Mean	Supervised Rehabilitation SD	Supervised Rehabilitation Total	Weight	Std. Mean Difference IV, Random, 95% CI
**1.7.1 Early approach**								
Abbott, 2010	64.7	27	54	76.9	27.6	53	22.6%	−0.44 [−0.83, −0.06]
**Subtotal (95% CI)**			**54**			**53**	**22.6%**	**−0.44 [−0.83, −0.06]**
Heterogeneity: Not applicable								
Test for overall effect: Z = 2.27 (P = 0.02)								
**1.7.2 Late approach**								
McGregor, 2011	62.92	24.79	161	71.49	24.98	177	48.3%	−0.34 [−0.56, −0.13]
Paulsen, 2020 A	0.79	0.17	73	0.8	0.22	73	29.1%	−0.05 [−0.38, 0.27]
**Subtotal (95% CI)**			**234**			**250**	**77.4%**	**−0.22 [−0.51, 0.06]**
Heterogeneity: Tau^2 = 0.02; Chi^2 = 2.18, df = 1 (P = 0.14); I^2 = 54%								
Test for overall effect: Z = 1.55 (P = 0.12)								
**Total (95% CI)**			**288**			**303**	**100.0%**	**−0.28 [−0.49, −0.07]**
Heterogeneity: Tau^2 = 0.01; Chi^2 = 2.95, df = 2 (P = 0.23); I^2 = 32%								
Test for overall effect: Z = 2.65 (P = 0.008)								
Test for subgroup differences: Chi^2 = 0.82, df = 1 (P = 0.36), I^2 = 0%								

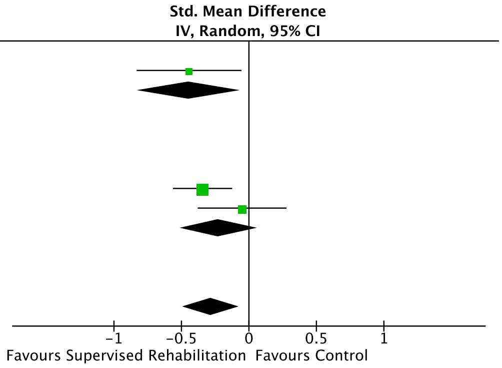

Figure S2. Pooled results from the subgroup analysis on the timing of supervised rehabilitation initiation for quality of life following lumbar spine surgery, comparing supervised physiotherapy to control groups (including unsupervised rehabilitation, educational interventions, or no treatment). Subgroup analysis reports results of: Early Approach (starting supervised rehabilitation within 4 weeks from surgery) and Late Approach (starting after 4 weeks from surgery). **(a)** 6 months after surgery **(b)** 1 year after surgery.

(a)

Study or Subgroup	Supervised Rehabilitation Mean	SD	Total	Control Mean	SD	Total	Weight	Std. Mean Difference IV, Random, 95% CI
**1.3.1 Early approach**								
Abbott, 2010	33.9	10.1	53	44	11.1	54	27.9%	−0.94 [−1.34, −0.54]
Janssens, 2016	43	5	12	38	5	13	23.9%	0.97 [0.13, 1.80]
**Subtotal (95% CI)**			**65**			**67**	**51.8%**	**−0.03 [−1.90, 1.85]**
Heterogeneity: Tau² = 1.71; Chi² = 16.30, df = 1 (P < 0.0001); I² = 94%								
Test for overall effect: Z = 0.03 (P = 0.98)								
**1.3.2 Late approach**								
Demir, 2014	19.5	3.33	22	17.5	6.07	22	26.3%	0.40 [−0.20, 1.00]
Rushton, 2015	37.88	7.28	8	38	13.22	7	21.9%	−0.01 [−1.03, 1.00]
**Subtotal (95% CI)**			**30**			**29**	**48.2%**	**0.30 [−0.22, 0.81]**
Heterogeneity: Tau² = 0.00; Chi² = 0.47, df = 1 (P = 0.49); I² = 0%								
Test for overall effect: Z = 1.12 (P = 0.26)								
**Total (95% CI)**			**95**			**96**	**100.0%**	**0.07 [−0.87, 1.01]**
Heterogeneity: Tau² = 0.78; Chi² = 24.36, df = 3 (P < 0.0001); I² = 88%								
Test for overall effect: Z = 0.15 (P = 0.88)								
Test for subgroup differences: Chi² = 0.10, df = 1 (P = 0.75), I² = 0%								

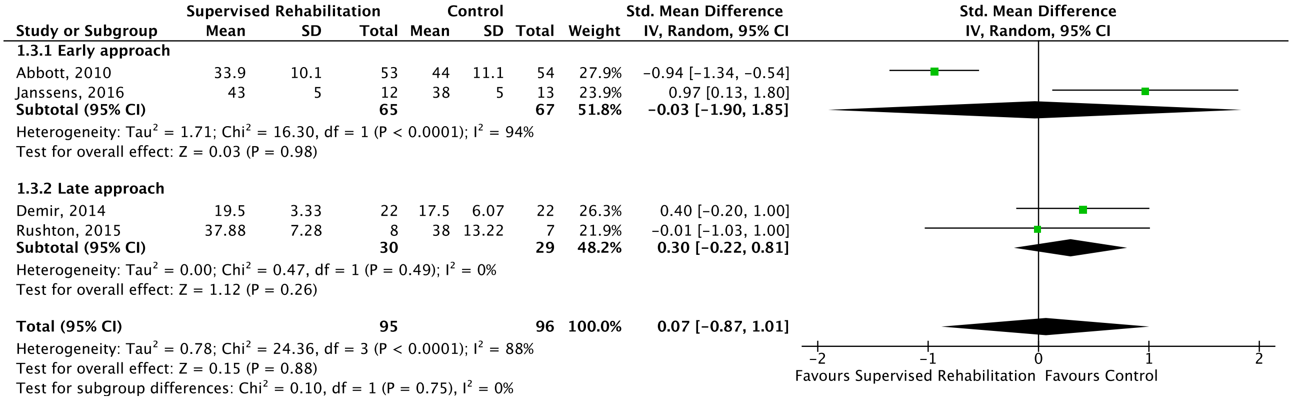

(b)

Study or Subgroup	Supervised Rehabilitation Mean	SD	Total	Control Mean	SD	Total	Weight	Std. Mean Difference IV, Random, 95% CI
**1.8.1 Early approach**								
Abbott, 2010	30.3	10.8	53	43.3	10.8	54	21.1%	−1.20 [−1.61, −0.78]
Janssens, 2016	36	2	12	38	5	13	15.3%	−0.50 [−1.30, 0.30]
Jentoft, 2020	24.4	5.28	20	27.5	10.79	31	18.8%	−0.34 [−0.90, 0.23]
**Subtotal (95% CI)**			**85**			**98**	**55.2%**	**−0.72 [−1.32, −0.12]**
Heterogeneity: Tau² = 0.19; Chi² = 6.57, df = 2 (P = 0.04); I² = 70%								
Test for overall effect: Z = 2.34 (P = 0.02)								
**1.8.2 Late approach**								
Ilves, 2017B	30.9	7.93	48	30.9	7.77	50	21.4%	0.00 [−0.40, 0.40]
McGregor, 2011	12.48	7	163	12.13	6.64	148	23.4%	0.05 [−0.17, 0.27]
**Subtotal (95% CI)**			**211**			**198**	**44.8%**	**0.04 [−0.16, 0.23]**
Heterogeneity: Tau² = 0.00; Chi² = 0.05, df = 1 (P = 0.83); I² = 0%								
Test for overall effect: Z = 0.39 (P = 0.69)								
**Total (95% CI)**			**296**			**296**	**100.0%**	**−0.38 [−0.89, 0.13]**
Heterogeneity: Tau² = 0.27; Chi² = 28.96, df = 4 (P < 0.00001); I² = 86%								
Test for overall effect: Z = 1.47 (P = 0.14)								
Test for subgroup differences: Chi² = 5.52, df = 1 (P = 0.02), I² = 81.9%								

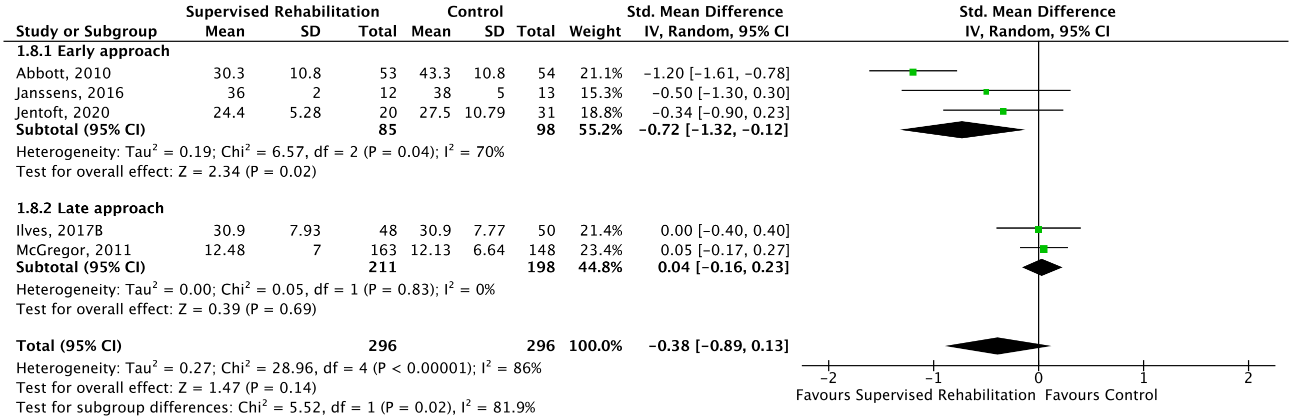

Figure S3. Pooled results from the subgroup analysis on the timing of supervised rehabilitation initiation for fear avoidance belief following lumbar spine surgery, comparing supervised physiotherapy to control groups (including unsupervised rehabilitation, educational interventions, or no treatment). Subgroup analysis reports results of: Early Approach (starting supervised rehabilitation within 4 weeks from surgery) and Late Approach (starting after 4 weeks from surgery). **(a)** 6 months after surgery **(b)** 1 year after surgery.

(a)

Study or Subgroup	Supervised Rehabilitation Mean	SD	Total	Control Mean	SD	Total	Weight	Std. Mean Difference IV, Fixed, 95% CI
**1.4.1 Early approach**								
Abbott, 2010	7.4	7.6	52	9.9	7.8	53	100.0%	−0.32 [−0.71, 0.06]
**Subtotal (95% CI)**			**52**			**53**	**100.0%**	**−0.32 [−0.71, 0.06]**
Heterogeneity: Not applicable								
Test for overall effect: Z = 1.64 (P = 0.10)								
**Total (95% CI)**			**52**			**53**	**100.0%**	**−0.32 [−0.71, 0.06]**
Heterogeneity: Not applicable								
Test for overall effect: Z = 1.64 (P = 0.10)								
Test for subgroup differences: Not applicable								

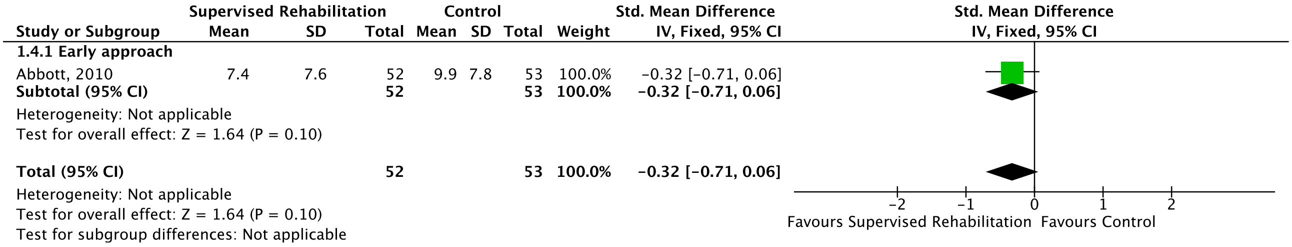

(b)

Study or Subgroup	Supervised Rehabilitation Mean	SD	Total	Control Mean	SD	Total	Weight	Std. Mean Difference IV, Fixed, 95% CI
**1.9.1 Early approach**								
Abbott, 2010	7.1	7.7	52	8	8.1	53	100.0%	−0.11 [−0.50, 0.27]
**Subtotal (95% CI)**			**52**			**53**	**100.0%**	**−0.11 [−0.50, 0.27]**
Heterogeneity: Not applicable								
Test for overall effect: Z = 0.58 (P = 0.56)								
**Total (95% CI)**			**52**			**53**	**100.0%**	**−0.11 [−0.50, 0.27]**
Heterogeneity: Not applicable								
Test for overall effect: Z = 0.58 (P = 0.56)								
Test for subgroup differences: Not applicable								

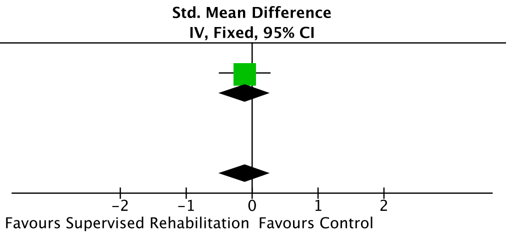

Figure S4. Pooled results from the subgroup analysis on the timing of supervised rehabilitation initiation for catastrophizing following lumbar spine surgery, comparing supervised physiotherapy to control groups (including unsupervised rehabilitation, educational interventions, or no treatment). Subgroup analysis reports results of: Early Approach (starting supervised rehabilitation within 4 weeks from surgery) and Late Approach (starting after 4 weeks from surgery). **(a)** 6 months after surgery **(b)** 1 year after surgery.

(a)

Study or Subgroup	Control Mean	Control SD	Control Total	Supervised Rehabilitation Mean	Supervised Rehabilitation SD	Supervised Rehabilitation Total	Weight	Std. Mean Difference IV, Fixed, 95% CI
**1.10.1 Early approach**								
Abbott, 2010	33.7	8.1	54	43.3	10.3	53	100.0%	−1.03 [−1.43, −0.63]
**Subtotal (95% CI)**			**54**			**53**	**100.0%**	**−1.03 [−1.43, −0.63]**
Heterogeneity: Not applicable								
Test for overall effect: Z = 4.99 (P < 0.00001)								
**Total (95% CI)**			**54**			**53**	**100.0%**	**−1.03 [−1.43, −0.63]**
Heterogeneity: Not applicable								
Test for overall effect: Z = 4.99 (P < 0.00001)								
Test for subgroup differences: Not applicable								

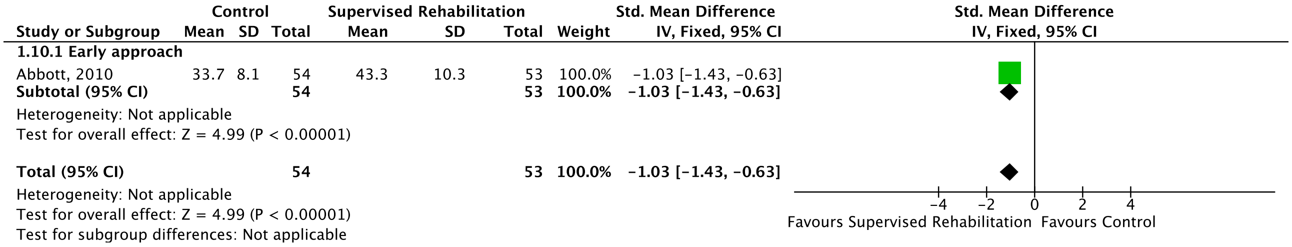

(b)

Study or Subgroup	Usual Care Mean	Usual Care SD	Usual Care Total	Supervised Rehabilitation Mean	Supervised Rehabilitation SD	Supervised Rehabilitation Total	Weight	Std. Mean Difference IV, Fixed, 95% CI
**1.5.1 Early approach**								
Abbott, 2010	32.5	6.8	54	41.2	8.4	53	100.0%	−1.13 [−1.54, −0.72]
**Subtotal (95% CI)**			**54**			**53**	**100.0%**	**−1.13 [−1.54, −0.72]**
Heterogeneity: Not applicable								
Test for overall effect: Z = 5.42 (P < 0.00001)								
**Total (95% CI)**			**54**			**53**	**100.0%**	**−1.13 [−1.54, −0.72]**
Heterogeneity: Not applicable								
Test for overall effect: Z = 5.42 (P < 0.00001)								
Test for subgroup differences: Not applicable								

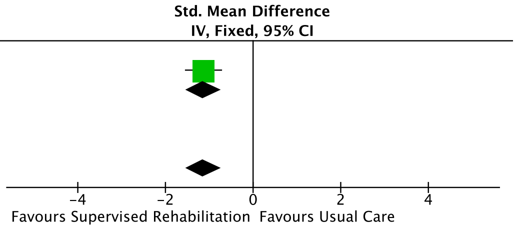

Figure S5. Pooled results from the subgroup analysis on the timing of supervised rehabilitation initiation for self-efficacy following lumbar spine surgery, comparing supervised physiotherapy to control groups (including unsupervised rehabilitation, educational interventions, or no treatment). Subgroup analysis reports results of: Early Approach (starting supervised rehabilitation within 4 weeks from surgery) and Late Approach (starting after 4 weeks from surgery). **(a)** 6 months after surgery **(b)** 1 year after surgery.

(a)

Study or Subgroup	Supervised Rehabilitation Mean	SD	Total	Control Mean	SD	Total	Weight	Std. Mean Difference IV, Random, 95% CI
**1.11.1 Late approach**								
Dolan, 2000	7	3	9	8	5	11	19.5%	−0.23 [−1.11, 0.66]
McGregor, 2011	9.45	2.7	182	8.63	2.1	137	80.5%	0.33 [0.11, 0.56]
**Subtotal (95% CI)**			**191**			**148**	**100.0%**	**0.22 [−0.21, 0.66]**
Heterogeneity: $Tau^2 = 0.05$; $Chi^2 = 1.44$, df = 1 (P = 0.23); $I^2 = 31\%$								
Test for overall effect: Z = 1.01 (P = 0.31)								
**Total (95% CI)**			**191**			**148**	**100.0%**	**0.22 [−0.21, 0.66]**
Heterogeneity: $Tau^2 = 0.05$; $Chi^2 = 1.44$, df = 1 (P = 0.23); $I^2 = 31\%$								
Test for overall effect: Z = 1.01 (P = 0.31)								
Test for subgroup differences: Not applicable								

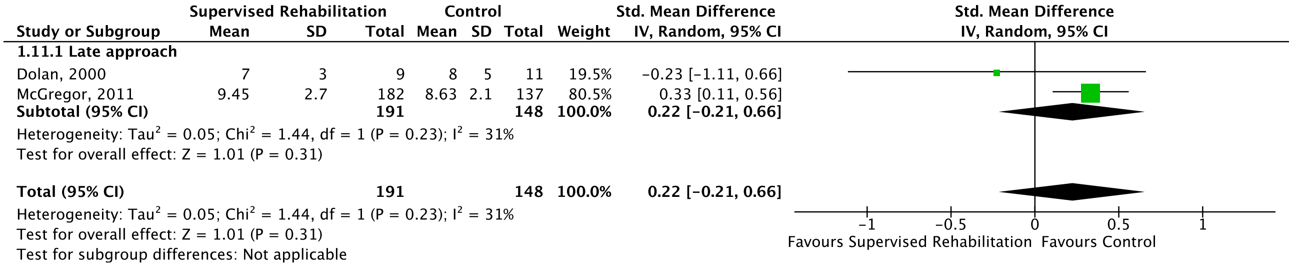

(b)

Study or Subgroup	Supervised Rehabilitation Mean	SD	Total	Control Mean	SD	Total	Weight	Mean Difference IV, Fixed, 95% CI
**1.12.1 Late approach**								
McGregor, 2011	7.2	2.6	156	6.83	2.82	137	100.0%	0.37 [−0.25, 0.99]
**Subtotal (95% CI)**			**156**			**137**	**100.0%**	**0.37 [−0.25, 0.99]**
Heterogeneity: Not applicable								
Test for overall effect: Z = 1.16 (P = 0.25)								
**Total (95% CI)**			**156**			**137**	**100.0%**	**0.37 [−0.25, 0.99]**
Heterogeneity: Not applicable								
Test for overall effect: Z = 1.16 (P = 0.25)								
Test for subgroup differences: Not applicable								

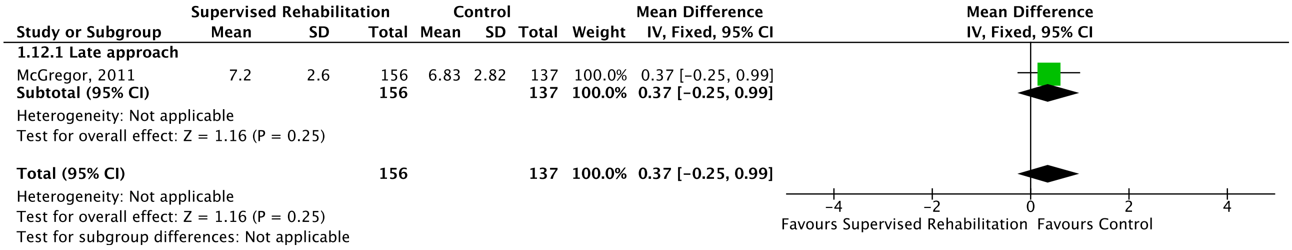

Figure S6. Pooled results from the subgroup analysis on the timing of supervised rehabilitation initiation for depression and anxiety following lumbar spine surgery, comparing supervised physiotherapy to control groups (including unsupervised rehabilitation, educational interventions, or no treatment). Subgroup analysis reports results of: Early Approach (starting supervised rehabilitation within 4 weeks from surgery) and Late Approach (starting after 4 weeks from surgery). **(a)** depression 1 year after surgery **(b)** anxiety 1 year after surgery.

(a)

Study or Subgroup	Supervised Rehabilitation Events	Supervised Rehabilitation Total	Control Events	Control Total	Weight	Risk Ratio M-H, Random, 95% CI
**1.6.1 Early approach**						
Abbott, 2010	16	41	25	40	41.7%	0.62 [0.40, 0.98]
Erdogmus, 2007	7	40	10	40	16.1%	0.70 [0.30, 1.66]
**Subtotal (95% CI)**		**81**		**80**	**57.8%**	**0.64 [0.43, 0.95]**
Total events	23		35			
Heterogeneity: Tau² = 0.00; Chi² = 0.06, df = 1 (P = 0.81); I² = 0%						
Test for overall effect: Z = 2.19 (P = 0.03)						
**1.6.2 Late approach**						
Paulsen, 2020 B	26	73	22	73	40.1%	1.18 [0.74, 1.88]
Rushton, 2015	1	8	1	7	2.1%	0.88 [0.07, 11.54]
**Subtotal (95% CI)**		**81**		**80**	**42.2%**	**1.17 [0.74, 1.85]**
Total events	27		23			
Heterogeneity: Tau² = 0.00; Chi² = 0.05, df = 1 (P = 0.82); I² = 0%						
Test for overall effect: Z = 0.67 (P = 0.50)						
**Total (95% CI)**		**162**		**160**	**100.0%**	**0.83 [0.57, 1.21]**
Total events	50		58			
Heterogeneity: Tau² = 0.04; Chi² = 3.91, df = 3 (P = 0.27); I² = 23%						
Test for overall effect: Z = 0.99 (P = 0.32)						
Test for subgroup differences: Chi² = 3.78, df = 1 (P = 0.05), I² = 73.6%						

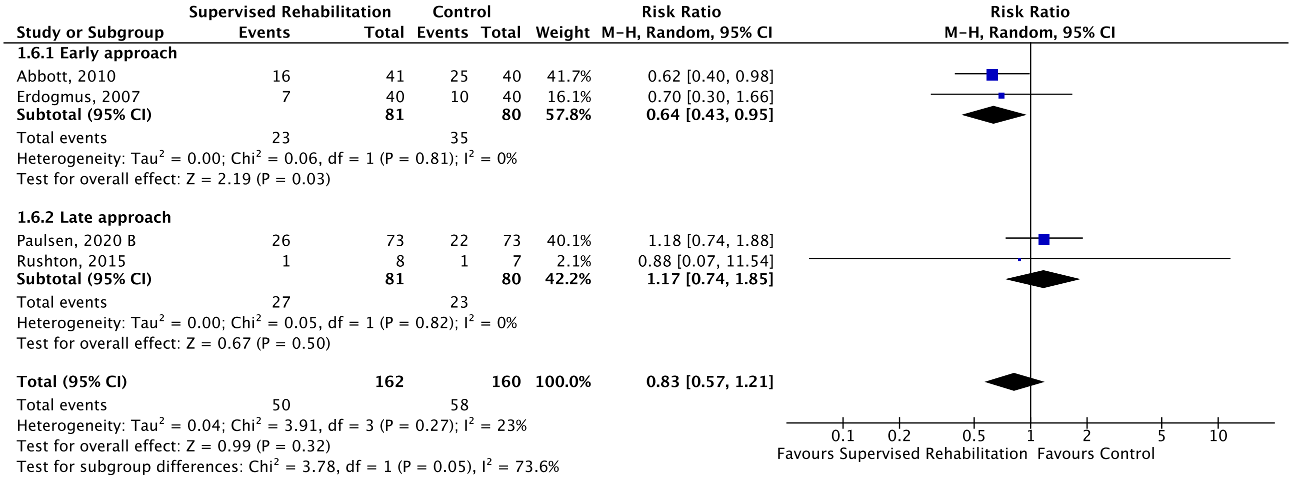

(b)

Study or Subgroup	Control Events	Control Total	Supervised Rehabilitation Events	Supervised Rehabilitation Total	Weight	Odds Ratio M-H, Fixed, 95% CI
**1.13.1 Late approach**						
Paulsen, 2020 B	16	73	19	73	100.0%	0.80 [0.37, 1.71]
**Subtotal (95% CI)**		**73**		**73**	**100.0%**	**0.80 [0.37, 1.71]**
Total events	16		19			
Heterogeneity: Not applicable						
Test for overall effect: Z = 0.58 (P = 0.56)						
**Total (95% CI)**		**73**		**73**	**100.0%**	**0.80 [0.37, 1.71]**
Total events	16		19			
Heterogeneity: Not applicable						
Test for overall effect: Z = 0.58 (P = 0.56)						
Test for subgroup differences: Not applicable						

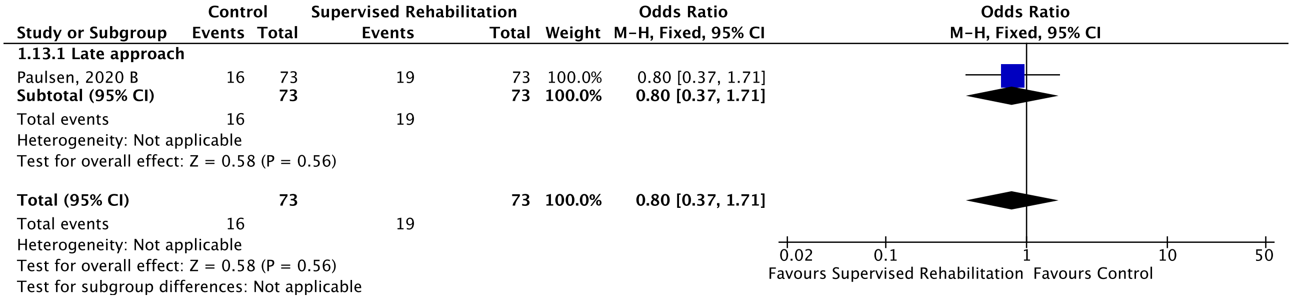

Figure S7. Pooled results from the subgroup analysis on the timing of supervised rehabilitation initiation for depression and anxiety following lumbar spine surgery, comparing supervised physiotherapy to control groups (including unsupervised rehabilitation, educational interventions, or no treatment). Subgroup analysis reports results of: Early Approach (starting supervised rehabilitation within 4 weeks from surgery) and Late Approach (starting after 4 weeks from surgery). **(a)** 6 months after surgery **(b)** 1 year after surgery.
